# A nerve-like self-healable conductive wire

**DOI:** 10.1093/nsr/nwae139

**Published:** 2024-04-18

**Authors:** Xuemei Fu, Benjamin C K Tee

**Affiliations:** Department of Materials Science and Engineering, National University of Singapore, Singapore; Institute for Health Innovation and Technology, National University of Singapore, Singapore; Department of Materials Science and Engineering, National University of Singapore, Singapore; Institute for Health Innovation and Technology, National University of Singapore, Singapore

In wearables and robotics, it is often required to deploy electronic devices at different spatially distributed locations for multi-functionality [1–3]. Thus, conductive wires for efficient interconnection of various electronic devices are desired. However, in practical applications, the frequent motion of wearers and robots will result in high-strain deformations of these wires, which could cause irreparable damage. Self-healing thus offers a promising solution to address these structural damages [4]. Such materials can autonomously recover their mechanical and electrical properties via dynamic bonds.

To-date, a variety of self-healable wires have been developed with remarkable properties. However, their relatively low tensile strength (0.05‒11 MPa) and poor dynamic stability, i.e. severe fluctuation in electrical resistances ranging from hundreds to thousands of ohms under dynamic conditions, have hindered their real-world applications in wearables and robotics [5]. Particularly, in terms of wearable healthcare applications, which require highly reliable and precise signal capture under various dynamic conditions, the poor dynamic stability of these wires can hardly meet the requirements.

**Figure 1.**
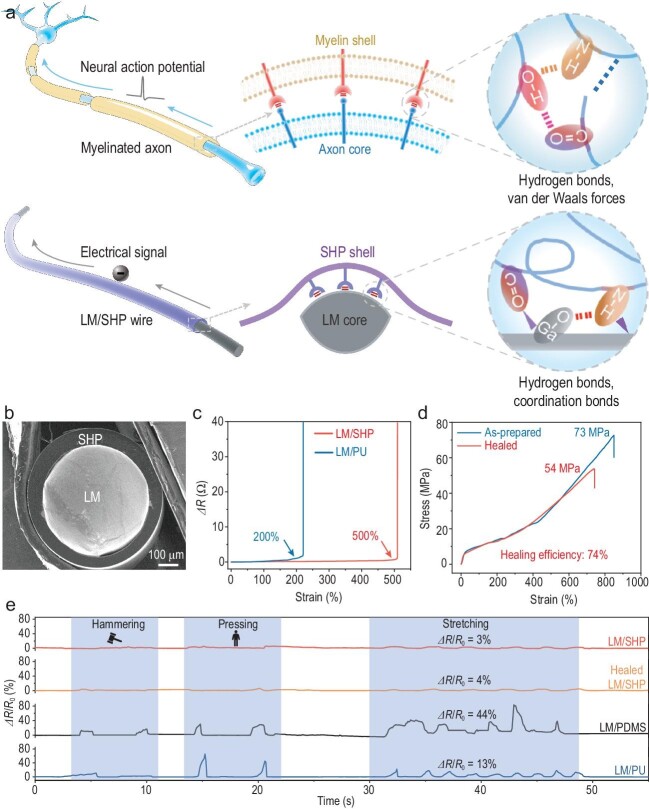
Core-shell structured, strong, self-healable conductive wire. (a) Schematics of the core-shell structured myelinated axon and LM/SHP wire. The top illustrates the van der Waals force and hydrogen-bond interactions between the myelin shell and axon core. The bottom illustrates the hydrogen and coordination-bond interactions between the SHP shell and LM core. (b) Cross-sectional microscopic morphology of LM/SHP wire. (c) Electrical resistance changes of different wires under increasing uniaxial strain. (d) Tensile test results of pristine and cut-and-healed LM/SHP wires. (e) Relative resistance changes of different wires under dynamic conditions. Reproduced with permission from ref. [6].

Inspired by the myelinated axon of nerve cells, Sun *et al.* pioneered the field of self-healable electronics by innovating a new family of dynamically stable self-healable wires based on a mechanical–electrical coupling strategy (Fig. 1a) [6]. These core-shell wires were composed of a GaInSn liquid metal (LM) core protected by a self-healable polymer (SHP) shell comprising acyl-semicarbazide (ASC) moieties and poly(1,4-butylene adipate) (Fig. 1b). Beyond the structural bionics, the interfacial interactions (hydrogen and coordination bonds) between the LM core and SHP shell were constructed by mimicking the interfacial interactions (hydrogen bonds and van der Waals forces) between the axon and myelin. The resulting wires (abbreviated as LM/SHP wires) presented unparalleled mechanical, electrical, and dynamic stability performance, for example, high electrical conductivities of 9.8 × 10^4^ S/m which remained stable under high strains up to 500% (Fig. 1c), a maximum tensile strength of 73 MPa at a high strain of 850%, and a self-healing efficiency of 74% in tensile strength after healing at 110°C for 12 h (Fig. 1d).

The outstanding performance of LM/SHP wires can be attributed to rich N–H and C=O groups in SHP, which offer a mechanical–electrical coupling effect through the strong interaction with gallium(III) oxide skin and gallium cations of LM, including the hydrogen bonds between N–H groups and gallium(III) oxide and the coordination bonds between C=O, N–H groups and gallium cations. In contrast, common stretchable polymers such as polydimethylsiloxane (PDMS) and polyurethane (PU) cannot provide strong bonds with LM. Therefore, the LM/SHP wires demonstrated enhanced electrical stability under diverse dynamic conditions, e.g. hammering, pressing, and stretching, which outperformed similar wires based on PDMS and PU (Fig. 1e). The high dynamic stability enabled precise monitoring of health status and daily activities, such as in simulated cases of limb tremors caused by Parkinson's disease.

The proposed LM/SHP wires, with their unique combination of high strength, self-healing, stretchability and conductivity, can afford higher levels of robustness that can greatly broaden their applications in wearable healthcare devices, intelligent robotics, and implantable electronics. Furthermore, the core or shell layer of these wires can be combined with other functional materials to incorporate more functions [7]. The high self-healing temperature (110°C) presents some limitations, which needs to be further reduced to ensure the thermal safety of the other electronic components.
